# Special Issue: Emerging Approaches for the Performance Assessment and Prediction of Cement-Based Materials

**DOI:** 10.3390/ma16216974

**Published:** 2023-10-31

**Authors:** Nima Farzadnia, Amin Malek

**Affiliations:** 1 Department of Civil, Geological, and Environmental Engineering, College of Engineering and Mines, University of Alaska Fairbanks, Fairbanks, AK 99775, USA; 2 Department of Computer and Electrical Engineering, California State University, Bakersfield, CA 93311, USA

The current Special Issue, entitled “Emerging Approaches for Performance Assessment and Prediction of Cement-Based Materials”, aims to showcase cutting-edge research into the technologies, smart sensing systems, and tools for assessing and predicting the performance of cement-based materials. Special emphasis is placed on understanding innovative strategies for characterizing the microstructure and durability of these materials, as well as harnessing smart sensing systems and data-driven approaches, all of which hold the potential to redefine our modern infrastructure.

Cement-based materials have always been the backbone of our construction industry, providing strength, durability, and resilience to our built environment. Over the years, researchers have delved deep into understanding the intricate physicochemical processes that govern the behavior of these materials, studying hydration processes, the formation of various crystalline and amorphous phases, and interactions with admixtures as well as deterioration mechanisms [1,2,3]. The advent of smart sensing systems has added a new dimension to this research, allowing for the real-time monitoring of stress, strain, and environmental impacts on concrete structures, thereby enabling timely interventions [4]. Furthermore, data-driven approaches, bolstered by the rise of machine learning and artificial intelligence, are beginning to play a pivotal role in predicting the long-term performance of concrete structures. These methodologies not only rely on historical data but also incorporate real-time feedback from sensors, leading to highly accurate and dynamic predictive models [5].

The research scope of this Special Issue encompasses, but is not limited to, advanced imaging techniques for cement microstructure; novel experimental and computational methodologies for durability assessments; predictive modeling using data analytics; and the integration and utility of smart sensing systems in cement-based constructs. Additionally, leveraging the power of big data to develop high-performance composites with better properties and enhanced performance, remains the focal point of this Special Issue.
